# Concurrency and other sexual partnership patterns reported in a survey of young people in rural Northern Tanzania

**DOI:** 10.1371/journal.pone.0182567

**Published:** 2017-08-24

**Authors:** Aoife M. Doyle, Mary L. Plummer, Helen A. Weiss, John Changalucha, Deborah Watson-Jones, Richard J. Hayes, David A. Ross

**Affiliations:** 1 MRC Tropical Epidemiology Group, London School of Hygiene & Tropical Medicine, London, United Kingdom; 2 Independent consultant, Dar es Salaam, Tanzania; 3 National Institute for Medical Research, Mwanza, Tanzania; 4 Department of Clinical Research, London School of Hygiene & Tropical Medicine, London, United Kingdom; 5 World Health Organization, Geneva, Switzerland; Indiana University, UNITED STATES

## Abstract

African adolescents and young adults remain at substantial risk of infection with HIV and other sexually transmitted infections (STIs), and AIDS is the leading cause of death among African adolescents (10–19 years). Sexual partnership patterns influence transmission risk of sexually transmitted infections. We describe patterns reported by youth (15-30y) in a community-based survey in Tanzania. Among participants reporting multiple partners, we investigated factors associated with reported concurrency. Female (N = 6513) and male (N = 7301) participants had median ages of 21 and 22 years, respectively. Most participants (92%) reported having previously been sexually active, of whom 15% of males and <1% of females reported ≥4 partners in the past year. The point prevalence of concurrency was 2.3% (95%CI 1.9–2.9) for females and 10.6% (95%CI 9.3–12.1) for males. High levels of multiple and concurrent partnerships were reported by those previously married. Females were more likely than males to report having spousal/regular partners and longer partnership lengths. Compared to males, the partnerships reported by females were less likely to be new partnerships, and more likely to be defined by the respondent as still ‘ongoing’. Females reporting younger sexual debut were more likely to report concurrent sexual partners. Far fewer young women reported multiple and concurrent partnerships, but we cannot definitively conclude that concurrency was uncommon for women, because stigma towards women’s multiple sexual partnerships might contribute to substantial under-reporting, as was found in extensive qualitative research in the study population. This study provides one of the most comprehensive quantitative descriptions of partnership patterns of young people in an African setting. Interventions addressing sexual risk among youth should involve male partners, empower women to protect themselves within different types of partnerships, and encourage a greater openness about young people’s sexual relationships.

## Introduction

African adolescents and young adults remain at substantial risk of infection with HIV and other sexually transmitted infections (STIs), and AIDS is the leading cause of death among African adolescents (10–19 years)[1]. Mortality from HIV is decreasing in all age groups except adolescents[2]. Having unprotected sex with multiple partners is one of the key risk factors for HIV in sub-Saharan Africa[3].

Risk of HIV/STI acquisition is difficult to estimate at one timepoint for one partnership, and even more challenging to assess over time in multiple fluid relationships. Reported number of partners is often used as a crude indicator of risk of HIV infection. However, not all sexual partnerships carry the same risk of infection and STI/HIV transmission at the partnership level is influenced by individual characteristics, partner characteristics (e.g number of partners’ partners and whether they are concurrent (overlap in time), duration of infection in the partners)), and partnership dynamics/characteristics (e.g. timing and duration of partnerships, frequency of sexual encounters, consistency of condom use) [4–11]. High levels of concurrency within a population create interconnected sexual networks that allow infection to spread more easily[12]. Concurrency does not increase the risk of infection to the person who has concurrent partners over and above having multiple partners but it can increase the risk of infection to his/her partners[13].

Studies show that in many parts of sub-Saharan Africa it is common for men to have concurrent sexual relationships[9, 14–17]. One factor contributing to this is the practice of polygyny (i.e. a man having multiple wives). In some areas, it is also not unusual for women to have concurrent partnerships, although their secondary relationships generally may involve fewer partners, longer relationship durations, and greater secrecy than men’s [9, 14–17].

Among young people in sub-Saharan Africa, qualitative research has provided important insights into concurrency. A key qualitative study took place in nine villages in Mwanza Region, Tanzania from 1999 to 2002. This study involved three person-years of participant observation, 184 formal in-depth interviews with HIV-positive and randomly selected youth, 29 focus group discussions with 50 follow-up individual interviews, and simulated patient exercises conducted in 19 health facilities[18]. That study reported that unmarried youth tended to have three types of sexual relationships: one-time encounters; those that involved occasional opportunistic encounters within an open-ended timeframe; and those that were steady, sometimes semi-public partnerships, which involved more frequent encounters. The vast majority of young women were sexually active by the time they left school, and it was common for those who were not married to have a steady sexual partner who they met regularly for sex. Sometimes, unmarried young women also engaged in overlapping, one-time encounters, or casual relationships involving occasional encounters. It also was not unusual for unmarried young men to have a series of partners in close succession or to maintain open-ended, concurrent relationships. Unmarried young people generally hid concurrent sexual partnerships, because discovery of infidelity could lead to relationship break-up. Young women were especially secretive for fear of broader social stigma if they were discovered. Approximately half of young married respondents reported they had married a partner within a month of first meeting their spouse. Extramarital relationships were not uncommon. Polygyny is legal in Tanzania, and polygynous marriages can be registered as either Muslim or traditional. However, the 1999–2002 qualitative study found that few marriages of any kind were legally registered in rural Mwanza, and many Christians were in polygynous marriages. Few young men had the resources, opportunity, and desire to have a polgynous marriage. A larger proportion of young women were in polygynous marriages which they had joined as junior wives.

Quantitative research on multiple sexual partnerships among young people has been limited. The sensitive nature of sexual partnerships makes accurate measurement of multiple partnerships and broader sexual networks very challenging. Even variables such as the approximate date when a sexual relationship began, or a person’s lifetime number of partners, can be problematic to measure correctly, particularly if respondents have poor numeracy, difficulty with recall, or concerns that their behaviours might be socially undesirable [19–21]. These problems are compounded when collecting more complex data, such as the durations of multiple sexual relationships, and the frequency of sex within each of them. Given these challenges, few measures have been developed to rigorously evaluate such variables, and few surveys have attempted to collect such data comprehensively. The few quantitative studies that have detailed sexual partnership patterns among young people in sub-Saharan Africa have found multiple and overlapping sexual partnerships to be frequently reported [22–27]. Quantitative descriptions of sexual partnerships among young people have often lacked details on the type of overlapping partners, the nature of concurrency, and the factors associated with it. Concurrency among young people remains poorly understood.

Data from a cluster randomised trial provided a unique opportunity to explore partnership patterns and concurrency in a large sample of young people in Tanzania. In this paper, we present an analysis of sexual partnership patterns for ~14,000 young people living in rural Mwanza in 2007/8. Specifically, we examine the characteristics of the individual sexual partnerships reported by the study participants, and the factors associated with concurrent partnerships in the past year. The 2007/8 survey was informed by, and built upon, qualitative research on young people’s sexual relationships conducted within the study area[18].

## Material and methods

### Study design

In 2007/8, interviews were conducted with respondents aged 15–30 years from the 20 communities that participated in the MEMA kwa Vijana cluster randomized trial in rural Mwanza, Tanzania[28, 29]. All of those who had attended at least one of school years 5–7 (i.e. the last 3 years of primary school) during the trial (1999–2002) were eligible to participate in this cross-sectional study. During a household census, 16,747 potentially eligible young people were invited to attend the survey[29]. Of those invited, 13,281 (79%) attended the survey along with 2426 young people who had not been invited. Of the 15,707 who attended the survey, 1,893 (12%) were not eligible to participate as either they had not attended an eligible school in the relevant years (n = 1,882), they did not provide consent (n = 8), or their registration information was missing (n = 3). A total of 13,814 eligible young people (7301 (53%) males and 6513 (47%) females) were interviewed between June 2007 and July 2008. Demographic and sexual behaviour data were collected using a face-to-face questionnaire administered by a same-sex interviewer. Blood and urine samples were also collected.

### Ethics and informed consent

The trial protocol received ethical and research clearance from the Tanzanian Medical Research Coordinating Committee (21^st^ Nov 2005) and the Ethics Committee of London School of Hygiene & Tropical Medicine (No. 3039, 2005). Signed informed consent was obtained from each participant on the day of the survey. Additional written consent from parents was obtained for participants under the age of 18 years.

### Measures

Participants in the 2007/8 survey responded to direct questions about their total number of sexual partners and/or new sexual partners in fixed periods of time (lifetime, past 12 months, past 4 weeks). Respondents were also asked more detailed questions on their 3 most recent sexual partners in the past year. Respondents were asked to classify each partner according to the following categories: spouse, other regular partner, casual partner, sex worker. Partnership patterns in the last 12 months (abstinent, single monogamous, serial monogamous, concurrent) were described based on the reported timing of first and last intercourse with the last 3 partners in the past 12 months. To promote recall and accuracy of reports, respondents had the option of reporting timings in days, weeks or months. Partnerships were excluded if reported timing of last sex with partner was ≥365 days, and if timing of first or last sex were missing. Participants were excluded from the partnership pattern analysis if at least one of their partnerships had missing information on the timing of first and last sex. In total 469 (3%) partnerships and 174 (1%) participants were excluded. Respondents reporting multiple partnerships with no overlap in time were defined as having a serial monogamous pattern, and respondents reporting at least two partnerships overlapping in time were considered to have a concurrent partnership pattern.

Using the timing of first and last intercourse with each partner, we calculated the following UNAIDS recommended indicators: point prevalence of concurrency at 6 months prior to interview, the proportion of those reporting multiple partnerships who reported partnerships that were concurrent, and the cumulative prevalence of concurrent partnerships (proportion who have any overlapping partnership in past year)[30]. UNAIDS recommend 6-month point prevalence because in most cases it will be clear whether the respondent had sex again with their partner and thus avoids asking the respondent to speculate as to whether they will have sex again with that partner[30]. We also calculated current concurrency, an alternative measure of the prevalence of concurrency that does not require the recall of timings of sex, as the proportion of respondents who reported that at least 2 of their partnerships were ‘ongoing’ at the time of the survey.

For participants who reported more than 3 partners in the previous 12 months, concurrent partnerships over this time period may have been missed as we only recorded the timing of the 3 most recent partnerships and often those 3 partnerships took place in the 6 months prior to the survey. Some authors have chosen to exclude those who report >3 partners in the past 12 months when looking at concurrency[22] as their inclusion may lead to an underestimate of concurrency. We have included these individuals when looking at partnership patterns, but have calculated the concurrency measures with and without these individuals.

Overall patterns of condom use, total frequency of sex, and average partnership length were calculated using information on the last 3 partners in the previous 12 months. Participants were categorized as being married if they reported that they were ‘married’ or ‘living as married’. A respondent was considered to be in a polygonous union if they reported that they had more than one wife (males) or reported that their husband had more than one wife (females).

### Laboratory analysis

Sera were tested for HIV in parallel, using the 3rd generation Murex HIV 1.2.O ELISA (Abbott-Murex, Dartford, UK) and the 3rd generation Vironostika HIV Uniform II plus O (bioMérieux, Boxtel, Netherlands). Sera with discordant or indeterminate ELISA results were retested up to two more times on both ELISAs. Persistently discordant samples were tested for P24 antigen using Biorad Genetic System HIV1 Ag EIA (Biorad, Lacoquette, France) and P24 negative samples were tested with the Inno-Lia HIV1/2 score Assay (Inno-Genetics NV, Gent, Belgium). Inno-Lia negative and indeterminate specimens were classified as negative. Sera were tested for antibodies to *Herpes simplex* virus type 2 (HSV2) using a type-specific Kalon IgG ELISA (Kalon Biologicals, Guildford, UK), according to the manufacturer’s instructions. Persistently indeterminate samples were classified as negative. Urine specimens were tested for *Chlamydia trachomatis* (CT) and *Neisseria gonorrhoeae* (NG) by Amplicor® PCR (Roche Diagnostics, Branchburg, USA). NG samples that remained positive on repeated Amplicor® PCR testing were confirmed with an in-house 16S rDNA PCR.

### Statistical analysis

Respondent patterns of sexual behaviour and HIV/STI prevalence were summarised by gender and marital status. Median age at first sex was calculated using survival techniques. Reported characteristics of partners and partnerships were summarised by gender of respondent and type of sexual partner. For some analyses, ‘other regular’ and ‘casual/sex worker’ partner types were grouped together as ‘non-spousal partners’ and/or analysis was conducted without stratification by gender. To account for the clustered sampling design (clustering by the 20 survey communities), survey commands in STATA 13 were used to calculate confidence intervals, and to compare proportions (Pearson chi-squared statistic), and compare means (adjusted Wald test).

Among those who reported the details of 2 or 3 partners in the past year, we investigated factors associated with point prevalence of concurrency. Analysis was restricted to those who reported multiple partners (≥2 partners) as we wanted to separate out the correlates of concurrency from those for engaging in multiple partnerships. Odds ratios were calculated using conditional logistic regression, conditioned on study community to allow for between-community variation, and p-values were calculated using the likelihood ratio test. Age and other demographic variables associated with concurrency on univariate analysis (p<0.10) were included in a multivariate model. A final demographic model included age and variables independently associated with concurrency in the multivariate model (p<0.10). Similarly, all sexual behaviour indicators found to be associated with concurrency on univariate analysis with p<0.10 were included in a multivariate model along with variables from the final demographic model. The presence of multicollinearity in the final model was explored by observing changes in the standard error of exposures when variables were removed from the model. Where multicollinearity between two independent variables was observed, the variable with the weakest association with concurrency was removed. Models were fitted separately for males and females.

## Results

### Survey participants

The median and interquartile age range (IQR) was 22 (20–24) years for males and 21 (19–23) years for females (**Table 1**). The majority (84%) were Christian, and 25% had reached secondary level of education or higher. A third of males (34%) and just over half (57%) of females reported being currently married. Notably, almost 10% of females already considered themselves divorced. Among those currently married, 2% of males and 13% of females reported being in a polygynous union, with 29 (0.8%) of females reporting that their husbands had more than 2 wives.

**Table 1:** Demographic and sexual behaviour characteristics and HIV/STI prevalence.

	Males (7301)		Females (6513)		p-value^*7*^
	n	(% or median (IQR))	n	(% or median (IQR))	
***Demographic characteristics***					
Median age (IQR)	7300	22 (20, 24)	6513	21 (19, 23)	P<0.001
Religion					P<0.001
*Christian*	5884	(80.7)	5764	(88.7)	
*Muslim*	330	(4.5)	278	(4.3)	
*None/other*	1076	(14.8)	456	(7.0)	
*Marital status*					P<0.001
*Never married*	4608	(63.3)	2192	(33.8)	
*Currently married*	2458	(33.8)	3689	(56.9)	
*Previously married*	215	(3.0)	608	(9.4)	
Polygynous union	47	(1.9)	464	(12.6)	P<0.001
Time slept away in past 12 months					P<0.001
*Never*	2335	(32.4)	3393	(52.4)	
*Up to 1 month*	3337	(46.3)	2116	(32.7)	
*1–3 months*	727	(10.1)	538	(8.3)	
*>3 months*	812	(11.3)	427	(6.6)	
*Highest level of education*					P<0.001
*Primary or less*	5097	(69.9)	5259	(80.9)	
*Secondary or higher*	2196	(30.1)	1244	(19.1)	
Attended school/university in past 12 months	1738	(23.8)	814	(12.5)	P<0.001
Good knowledge on HIV acquisition^*1*^	5069	(69.6)	4184	(64.4)	P<0.001
***Reported sexual behaviour***					
Ever sexually active	6638	(91.0)	6058	(93.1)	P<0.001
Median age first sex (IQR)	7261	17 (15, 19)	6497	17 (15, 18)	P<0.001
Median lifetime number of sexual partners (IQR)	7267	4 (2, 6)	6495	2 (1, 3)	P<0.001
Number of partners in the past 12 months ^*2*^					
Median (IQR)		1 (1, 3)		1 (1, 1)	P<0.001
0	841	(12.7)	370	(6.1)	P<0.001
1	2694	(40.7)	5021	(83.0)	
2	1358	(20.5)	510	(8.4)	
3	730	(11.0)	105	(1.7)	
4+	996	(15.1)	41	(0.7)	
Pattern of partnerships in past 12 months ^*3*^^*,*^^*4*^					P<0.001
*Never had sex*	656	(9.1)	452	(7.0)	
*Abstinent*	880	(12.2)	389	(6.0)	
*Single partner*	2727	(37.7)	4990	(77.4)	
*Multiple partners- no overlap*	1025	(14.2)	268	(4.2)	
*Multiple partners- some overlap*	1940	(26.8)	351	(5.4)	
> = 2 new sexual partners in past year^*2*^	2088	(36.1)	208	(3.7)	P<0.001
>1 partner in last 4 weeks^*2*^	965	(14.6)	129	(2.1)	P<0.001
Type of partners in past 12 months^*2*^^*,*^^*3*^^*,*^^*4*^^*,*^^*5*^					P<0.001
*single spouse*	1272	(22.4)	3543	(63.2)	
*single regular partner*	702	(12.3)	1071	(19.1)	
*single casual partner or sex worker*	753	(13.2)	376	(6.7)	
*multiple partners- spouse/regular*	247	(4.3)	142	(2.5)	
*multiple partners- casual or sex worker*	785	(13.8)	82	(1.5)	
*multiple partners- spouse/regular & casual/sex worker*	1932	(33.9)	395	(7.0)	
Average partnership length (days) (median (IQR)) ^*2*^^*,*^^*3*^^*,*^^*4*^^*,*^^*5*^	243	(50, 725)	1034	(395,1506)	P<0.001
Pattern of condom use at last sex with partners in past 12 months ^*2*^^*,*^^*3*^^*,*^^*4*^^*,*^^*5*^					P<0.001
*Did not use*	3175	(55.9)	4518	(81.1)	
*Used with at least one partner*	990	(17.4)	182	(3.3)	
*Used with all partners*	1511	(26.6)	873	(15.7)	
Total number of times had sex in past 4 weeks (median, IQR) ^*2*^^*,*^^*3*^^*,*^^*4*^^*,*^^*5*^^*,*^^*6*^		2 (0, 5)		2 (0, 4)	P<0.001
**HIV/STI prevalence**					
HIV prevalence	133/7260	(1.8)	262 /6475	(4.1)	P<0.001
HSV2 prevalence	1876 /7260	(25.8)	2682/6475	(41.4)	P<0.001
CT/NG prevalence	177 /7290	(2.4)	174 /6502	(2.7)	P = 0.36

Note: missing values not presented in the table. Percentages use available data as denominator.

^1^Based on correct response to the following 3 questions: Can HIV be caught by making love with someone? (yes), Can you catch HIV by sharing a plate of food with a HIV positive person?(No), Can a person who looks strong and healthy have HIV?(yes).

^2^Among those who report ever having had sex.

^3^Excludes 60 females & 66 males with incomplete partnership records, and 10 participants (3 female, 7 male) who did not answer the sexual behaviour questions.

^4^ Excludes partnerships where most recent sex with partner was reported to be > = 1 year ago.

^5^ Among N = 11301 (5609 females, 5692 males) who report detailed information on at least one eligible partnership in last 12 months.

^6^1032 (10%) participants were missing total frequency of sex in the past 4 weeks (83% of whom were currently married). Total frequency is sum of frequency of sex reported for up to 3 most recent partners.

^7^ For continuous variables the p-values were calculated for the comparison of means using the adjusted Wald test.

### Patterns of partnerships, sexual behaviour and STI prevalence

Overall, 12,696 (92%) respondents reported ever having had sex and the median reported age at first sex was 17 years (IQR 15–19 years) for both males and females (**Table 1**). Males reported a median of 4 lifetime sexual partners (IQR 2–6) while females reported a median of 2 partners (IQR 1–3). Among those reporting having ever had sex, the median number of partners in the year prior to the survey was 1 for both sexes. Of ever sexually active participants, 12.7% of males and 6.1% of females reported no partners in the year prior to the survey (p<0.001). The prevalence of HIV and HSV2 was 1.8% and 25.8% respectively for males, and 4.0% and 41.4% respectively for females (**Table 1**).

Data on the timing of their most recent sexual partnerships in the past year was available for 13,678 (99%) respondents. Of these, males consistently reported higher numbers of partners and rate of partner change (Table 1). For example, males were more likely than females to report having had multiple partners in the previous year (41% vs. 9.6%, p<0.001), more than 2 new partners in the previous year (36.1% vs. 3.7%, p<0.001), more than one partner in the previous 4 weeks (14.6% vs. 2.1%, p<0.001), sex with a casual partner or sex worker in the past year (60.9% vs. 15.2%, p<0.001), and a shorter average partnership length (1.3 vs. 3.0 years, p<0.001). Marital status was associated with reports of multiple partners in the past year. Among females, 12.6% of never married, 6.2% of married, and 34.4% of previously married participants reported more than one sexual partner in the previous year (p<0.001). Multiple partnerships in the past year were reported by 43.4% of never married males, 49.3% of currently married and 75.0% of previously married males (p<0.001).

### Estimates of concurrency

Estimates of concurrency were also greater for males than for females (**Table 2**). The 6-month point prevalence of concurrency was 10.6% for males and 2.3% for females, and the proportion of those with multiple partnerships in the last 12 months who reported overlapping partnerships was 65% for males and 57% for females (**Table 2**, p = 0.006). The 12-month cumulative prevalence of concurrency was 26.8% for males and 5.4% for females, and the prevalence of current concurrency (participant reports at least 2 of their partnerships are ‘ongoing’ at the time of the survey) was 9.5% for males and 2.1% for females (**Table 2**). Despite the similarity between the overall prevalence of 6-month point and current concurrency, only 41% of those who had concurrent partnerships at 6 months had current concurrent partnerships, and only 45% of those with current concurrent partnerships had concurrent partnerships at 6 months prior to the survey.

**Table 2:** Measures of concurrency.

	Males ^*1*^		Females ^*1*^		
	(7228)		(6450)		
	n	(%)	n	(%)	p-value
**6 month point prevalence of concurrency**	768	(10.6)	150	(2.3)	<0.001
*Among ever sexually active*	768/6512	11.7	150/5998	2.5	<0.001
*Among those reporting < = 3 partners in past 12 months*	768/6533	11.8	150/6428	2.3	<0.001
*Excluding ‘one-off’ partnerships*	702	9.7	149	2.3	<0.001
**Cumulative prevalence of concurrent partnerships in the last year (last 3 partners only)**	1940	(26.8)	351	(5.4)	<0.001
*Among those reporting < = 3 partners in past 12 months*	1940/6960	27.9	351/6442	5.4	<0.001
**Proportion of individuals with multiple partnerships in the last year (last 3 partners only) who report concurrency**	1940/2965	(65.4)	351/619	(56.7)	0.006
**Current concurrency**					
> = 2 partnerships where ‘still in a relationship and will have sex again’	690	(9.5)	133	(2.1)	<0.001
> = 2 partnerships where ‘still in a relationship and will have sex again’ OR ‘relationship not continuing, but might have sex again’	1253	(17.3)	185	(2.9)	<0.001
**Type of concurrent partners**					
Spouse/regular partners	240	(30.2)	59	(36.9)	0.29
Casual partners	110	(13.9)	15	(9.4)	
Spouse/regular and casual partner	444	(55.9)	86	(53.8)	

^1^Excludes 60 females & 66 males with incomplete partnership records, and 10 participants (3 female, 7 male) who did not answer the sexual behaviour questions.

Details were collected on the last 3 partners in the previous 12 months. Of the 1037 respondents who reported >4 partners in the past 12 months, 60% reported that at least two of their last 3 partnerships started within the 6 months prior to the survey. These 619 respondents could not have been defined as having concurrent partners at the 6-month time point. Among those who reported having had only 3 partners in the past 12 months, 10% reported all 3 partnerships to have started within the past 6 months. We tried varying the definition of 6-month point prevalence of concurrency, and all estimates were similar i.e. 10–12% for males, and 2.3–2.5% for females **(Table 2).** Of the concurrent partnerships at the 6-month point prior to the survey, 56% were overlapping regular and casual partners, 31% were overlapping regular partners, and 13% were overlapping causal partners (**Table 2**).

### Characteristics of partnerships

This analysis was based on 16,623 partnerships (in the past 12 months) reported by 11,301 participants. This represents 82% of all potential partnerships with 16% of partnerships missing because of the study design (participants were only asked about last 3 partners) and 2% missing due to non-response or because the participant was excluded due to an incomplete partnership history. Of these, 10,263 (62%) partnerships were reported by males. The characteristics of partnerships varied by gender **(Table 3).** Males characterised the majority of their partners as casual (55%) whereas females characterised the majority of their partners as spousal (61%). The median duration of all types of partnerships was shorter for males than females **(Table 3).** Males and females reported that 51% and 31%, respectively, of their casual/ sex worker partnerships lasted only 1 day. Males and females reported that 42% and 31% respectively of their casual/sex worker partners, and 28% and 8% respectively of all their partners were newly acquired (first sex within the 3 months prior to the survey) **(Table 3)**.

**Table 3:** Reported characteristics of partnerships according to gender and type of partner^1^.

	Partnerships reported by males (10,262)^2^			Partnerships reported by females (6360)		
	Spousal	Other regular	Casual/ Sex Worker	Spousal	Other regular	Casual/ Sex Worker
	(2474, 24%)	(2241, 22%)	(5547, 55%)	(3866, 61%)	(1465, 23%)	(1029, 16%)
Living with partner^3^	2382, 96.8%	48, 2.2%	14, 0.3%	3587, 93.0%	28, 1.9%	5, 0.5%
Length of partnership						
*< = 1 mth*	52, 2.1%	388, 17.3%	3623, 65.3%	17, 0.4%	102, 7.0%	387, 37.6%
*1mth-1yr*	598, 24.2%	979, 43.7%	1620, 29.2%	422, 10.9%	490, 33.4%	508, 49.4%
*1–3 yr*	1045, 42.2%	627, 28.0%	234, 4.2%	1408, 36.4%	630, 43.0%	106, 10.3%
*3+ years*	779, 31.5%	247, 11.0%	70, 1.3%	2019, 52.2%	243, 16.6%	28, 2.7%
Length of partnership in days (median (IQR))	729 (364, 1458)	269 (88, 705)	0 (0, 87)	1399(727, 1823)	546 (213, 1034)	86 (26, 213)
First sex with partner within last 3 months	122, 4.9%	384, 17.1%	2339, 42.2%	43, 1.1%	115, 7.8%	321, 31.2%
Reported status of the relationship						
*Still in relationship and will have sex again*	2410, 97.5%	1460, 65.3%	1304, 23.6%	3599, 93.3%	1244, 85.3%	519, 50.7%
*Relationship not continuing but might have sex again*	16, 0.6%	290, 13.0%	910, 16.5%	64, 1.7%	40, 2.7%	47, 4.6%
*Relationships has completely ended*	45, 1.8%	486, 21.7%	3307, 59.9%	194, 5.0%	175, 12.0%	457, 44.7%
Used condom with partner at last sex	157, 6.4%	1051, 47.0%	2605, 47.0%	188, 4.9%	604, 41.4%	394, 38.6%
Number of times had sex with partner in past 4 weeks^3^^,^^4^ (median (IQR))	5 (2, 9)	0 (0, 2)	0 (0, 1)	3 (1, 5)	0 (0, 2)	0 (0, 1)
Have had an occasion when did not want to make love with partner	1184, 47.9%	652, 29.1%	670, 12.1%	2601, 67.5%	735, 50.5%	348, 34.2%
Refused to have sex and managed to avoid having sex the last time they did not want to make love with partner	906, 76.7%	516, 79.5%	550, 83.1%	1717, 66.6%	567, 77.7%	261, 75.4%

^1^Within each gender, comparisons between partner types differed significantly (<0.001) for each variable.

^2^ Partnership type is missing for one partnership reported by a male participant.

^3^ Missing for 136 partnerships, 83 (61%) of which were casual partnerships.

^4^ 1175 partnerships (7% of total number of partnerships) were missing frequency of sex in the past 4 weeks, 934 (79%) of which were spousal partnerships.

**Fig 1:**
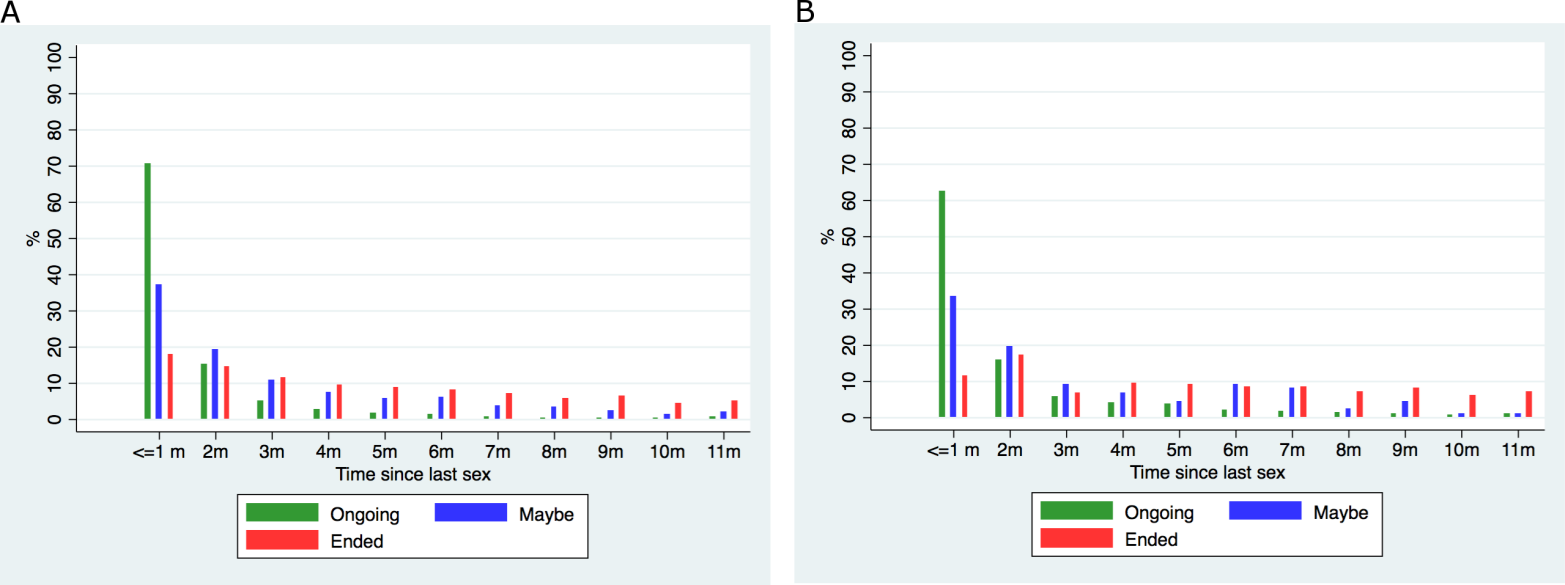
Reported time since last had sex according to relationship status^1^ of non-spousal partnerships reported in the past 12 months.^2^ 1 ‘Ongoing’ = still in a relationship and might have sex again; ‘Maybe’ = Relationship not continuing but might have sex again; ‘Ended’ = Relationship has completely ended. 2. Number of partnerships: Ongoing (males 2764, females 1763), Maybe (males 1200, females 87), Ended (males 3764, females 632).

Reporting on the status of their non-spousal regular partnerships, males said that 65% were still ‘ongoing’ and 13% had ‘ended but that they might make love again’. In comparison, females reported that 85% of non-spousal regular partnerships were ‘still ongoing’ and only 3% ‘ended but might make love again’. **Fig 1**shows the reported relationship status against the timing of last sex. For the majority of still ‘ongoing’ relationships, last sex was in the past month, and for those relationships which had ended but where further sexual intercourse was possible, the majority had last sex in the two months prior to the survey (**Fig 1**).

For 58% of partnerships reported by females and 24% of partnerships reported by males, the respondent reported that there had been an occasion when they did not want to make love with their partner. However, in only 70% of such partnerships reported by females and 79% of such partnerships reported by males had the respondent avoided having sex. Reported condom use at last sex was moderate for non-spousal partners and low for spousal partners. A higher frequency of sex was reported with spousal partners (**Table 3**).

### Characteristics of partners

Most spousal partners reported by males were younger (88%) and spousal partners reported by females were older (89%) (**Table 4**). Non-spousal partners reported by both genders were more likely than spousal partners to be the same age as the respondent (p<0.001), and more likely to have attended school or university in the previous 12 months (17% vs 0.2%, p<0.001) (**Table 4**). Fewer than 0.5% of spousal partners had attended school or university in the previous 12 months (**Table 4**).

**Table 4:** Reported characteristics of partners according to gender and type of partner^1^.

	Partnerships reported by males (10,262)^2^			Partnerships reported by females (6360)		
	Spousal	Other regular	Casual/ Sex Worker	Spousal	Other regular	Casual/ Sex Worker
	(2474, 24%)	(2241, 22%)	(5547, 55%)	(3866, 61%)	(1465, 23%)	(1029, 16%)
Age of partner relative to participant						
Older	31, 1.3%	118, 5.3%	537, 9.7%	3432, 88.8%	1141, 78.1%	686, 66.7%
Younger	2184, 88.3%	1473, 65.7%	3055, 55.2%	20, 0.5%	16, 1.1%	24, 2.3%
Same Age	247, 10.0%	620, 27.7%	1686, 30.4%	304, 7.9%	238, 16.3%	217, 21.1%
Not known	12, 0.5%	30, 1.3%	259, 4.7%	107, 2.8%	66, 4.5%	102, 9.9%
Median age difference in years between respondent and partner (IQR)^2^^**,**^^3^	3 (2, 5)	2 (0, 3)	1 (0, 3)	-4 (-6, -2)	-2 (-4, -1)	-2 (-4, 0)
Did partner complete primary school?						
Did not attend	365, 14.8%	122, 5.4%	338, 6.1%	297, 7.7%	32, 2.2%	35, 3.4%
No	605, 24.5%	223, 10.0%	585, 10.6%	651, 16.9%	58, 4.0%	49, 4.8%
Yes	1478, 59.8%	1779, 79.5%	3621, 65.4%	2875, 74.4%	1306, 89.3%	836, 81.3%
Not known	25, 1.0%	115, 5.1%	996, 18.0%	39, 1.0%	67, 4.6%	108, 10.5%
Partner attended school/university in last 12 months						
No	2469, 99.9%	1742, 77.8%	4675, 84.7%	3826, 99.7%	1179, 80.9%	900, 88.2%
Yes	2, 0.1%	497, 22.2%	842, 15.3%	13, 0.3%	279, 19.1%	120, 11.8%

^1^ Within each gender, comparisons between partner types differed significantly (<0.001) for each variable.

^2^ Partner type is missing for one partnership reported by a male participant.

^3^ Age difference was missing for 1399 (8%) partners, 803 (57%) of whom were casual partners.

### Factors associated with point prevalence of concurrency

In adjusted analysis, the point prevalence of concurrency (i.e. 6 months before the survey date) was associated with marital status, with currently married males more likely to report having concurrent partners compared to never married males (adjOR = 2.7; 95% CI 2.0, 3.5) (**Table 5**). No association with concurrency was seen for the other demographic/knowledge variables examined: age, religion, travel in the past 12 months, current school attendance, highest level of education, knowledge of HIV transmission (**Table 5**). Among females, a younger age at first sex, and among males a higher number of partners in the past year were associated with higher levels of concurrency. Respondents reporting that they had multiple regular partners were more likely to report concurrent partners than those who reported only casual partners or a mixture of regular and casual partners (**Table 5**).

**Table 5:** Factors associated with 6-month point prevalence of concurrency among those who reported 2 or 3 partners in the past 12 months.

	Males (1979)			Females (579)		
	Concurrency	Crude OR (95%CI)	Adjusted OR (95% CI)	Concurrency	Crude OR (95%CI)	Adjusted OR (95% CI)
Age group (years)		P<0.001	P = 0.15^2^		P = 0.54	P = 0.49^1^
*<21*	421, 16.2%	REF	REF	240, 20.4%	REF	REF
*21–22*	536, 21.8%	1.54 (1.10–2.15)	1.18 (0.83, 1.67)	171, 25.1%	1.33 (0.83–2.14)	1.27 (0.78, 2.06)
*23–24*	591, 24.5%	1.73 (1.25–2.40)	1.08 (0.76, 1.55)	125, 22.4%	1.17 (0.68–1.99)	1.11 (0.63, 1.94)
*25+*	431, 34.1%	2.81 (2.01–3.93)	1.46 (0.99, 2.14)	43, 27.9%	1.59 (0.75–3.37)	1.63 (0.74, 3.57)
Marital status		P<0.001	P<0.001^2^		P = 0.006	P = 0.007^1^
*Never married*	1107, 15.4%	REF	REF	191, 21.5%	REF	REF
*Currently married*	790, 37.0%	3.08 (2.46–3.84)	2.65 (2.03, 3.46)	209, 29.7%	1.61 (1.00–2.59)	1.4 (0.84, 2.3)
*Previously married*	77, 18.2%	1.25 (0.68–2.30)	1.10 (0.59, 2.04)	179, 16.2%	0.73 (0.43–1.26)	0.62 (0.35, 1.1)
Religion		P = 0.21	P = 0.87^2^		P = 0.27	P = 0.27^1^
*Christian*	1550, 23.1%	REF	REF	515, 21.9%	REF	REF
*Muslim*	91, 20.9%	0.84 (0.49–1.42)	0.87 (0.50, 1.50)	32, 25.0%	1.04 (0.44–2.43)	0.96 (0.40, 2.29)
*Other/none*	337, 29.4%	1.24 (0.95–1.63)	0.97 (0.73, 1.29)	32, 34.4%	2.00 (0.88–4.52)	2.02 (0.88, 4.67)
Length of time slept away in last 12 months		P = 0.53	P = 0.68^2^		P = 0.96	P = 0.94^1^
*None*	511, 25.6%	REF	REF	227, 23.3%	REF	REF
*up to 1 month*	1026, 23.9%	0.91 (0.71–1.17)	0.98 (0.76, 1.27)	204, 23.5%	1.05 (0.66–1.65)	1.03 (0.64, 1.64)
*1 month to 3 months*	205, 25.4%	0.96 (0.66–1.40)	1.21 (0.82, 1.79)	84, 22.6%	1.07 (0.58–1.97)	1.20 (0.64, 2.26)
*>3 months*	217, 18.9%	0.75 (0.50–1.11)	0.92 (0.61, 1.40)	60, 20.0%	0.87 (0.42–1.81)	0.95 (0.45, 2.01)
Attended school/university in last 12 months?		P<0.001	P = 0.52^2^		P = 0.05	P = 0.11^1^
*No*	1691, 25.8%	REF	REF	552, 23.4%	REF	REF
*Yes*	288, 14.2%	0.49 (0.34–0.70)	1.23 (0.66, 2.29)	27, 11.1%	0.34 (0.10–1.16)	0.39 (0.11, 1.39)
Highest level of education		P<0.001	P = 0.23^2^		P = 0.22	P = 0.90^1^
*Primary or less*	1571, 26.6%	REF	REF	527, 23.3%	REF	REF
*Secondary or higher*	408, 14.5%	0.48 (0.36–0.65)	0.72 (0.42, 1.24)	52, 17.3%	0.63 (0.30–1.35)	1.07 (0.39, 2.90)
Knowledge on HIV acquisition		P = 0.64	P = 0.61^2^		P = 0.41	P = 0.43^1^
*0–2 correct*	603, 25.5%	REF	REF	203, 24.6%	REF	REF
*3 correct*	1374, 23.5%	0.95 (0.75–1.19)	0.94 (0.74, 1.19)	374, 21.7%	0.84 (0.56–1.27)	0.84 (0.55, 1.29)
Age at first sex		P = 0.47	P = 0.85^4^		P = 0.02	P = 0.01^3^
*> = 16 yrs*	1399, 23.7%	REF	REF	345, 20.3%	REF	REF
*< 16yrs*	571, 25.0%	1.09 (0.87–1.37)	0.98 (0.76, 1.26)	234, 26.5%	1.60 (1.07–2.40)	1.74 (1.14, 2.65)
Lifetime number of sexual partners		P = 0.05	P = 0.69^4^		P = 0.50	P = 0.45^3^
*1–2*	141, 17.7%	0.76 (0.47–1.22)	0.89 (0.52, 1.51)	106, 17.9%	0.64 (0.36–1.15)	0.62 (0.34, 1.13)
*3–4*	711, 22.4%	REF	REF	292, 24.7%	REF	REF
*5–9*	789, 25.0%	1.17 (0.92–1.50)	0.86 (0.66, 1.13)	154, 22.1%	0.88 (0.54–1.41)	0.84 (0.51, 1.39)
*10+*	335, 28.4%	1.39 (1.03–1.87)	0.84 (0.60, 1.18)	27, 25.9%	0.96 (0.38–2.43)	0.87 (0.32, 2.34)
*Number of partners in the past 12 months*		P<0.001	P<0.001^4^		P = 0.24	P = 0.10^3^
*2*	1311, 20.3%	REF	REF	485, 21.9%	REF	REF
*3*	668, 31.6%	1.84 (1.49–2.29)	2.66 (2.09, 3.40)	94, 27.7%	1.37 (0.82–2.29)	1.60 (0.93, 2.77)
*Partner type*: *Regular*, *casual or both*		P<0.001	P<0.001^4^		P = 0.03	P = 0.07^3^
*Regular (including spouse)*	212, 62.7%	REF	REF	137, 31.4%	REF	REF
*Casual (including sex worker)*	537, 8.4%	0.06 (0.04–0.09)	0.06 (0.04, 0.10)	73, 15.1%	0.40 (0.19–0.86)	0.47 (0.21, 1.05)
*Both regular and casual*	1229, 24.3%	0.20 (0.15–0.27)	0.15 (0.11, 0.21)	369, 21.1%	0.61 (0.39–0.96)	0.60 (0.37, 0.96)

^1^Adjusted for age group, marital status, and whether attended school in past 12 months.

^2^ Adjusted for age group, marital status, attended school in last year and highest level of education.

^3^ Adjusted for age group, marital status, age at first sex and type of partners.

^4^ Adjusted for age group, marital status, number of partners in past year, type of partners.

## Discussion

This population-based study shows high levels of reported concurrency among young males in rural Tanzania, with relatively low levels of concurrency reported by young females. Few similar studies of partnership patterns have been carried out in young people in sub-Saharan Africa and most have focused only on males, have had relatively small sample sizes, or have only asked detailed questions on non-spousal partners. We asked respondents what the current relationship status was for each of their last 3 partners in the past year, which allowed us to examine the usefulness of a measure of current concurrency in this population.

Compared to females in our study, males reported a higher number of sexual partnerships, new partners, and concurrent partnerships. Males also reported higher levels of condom use, and partnerships of shorter duration than females, but reported coital frequency was similar in both genders. Compared to males, the partnerships reported by females were less likely to be new partnerships, and more likely to be defined by the respondent as still ‘ongoing’. These quantitative results are similar to findings of the in-depth qualitative research conducted within the same population from 1999–2002[18], and also to other research in sub-Saharan Africa[14, 22, 23]^,^[24, 25]. In South Africa, for example, young males were more likely than females to report having concurrent partners rather than serial partners[22, 31]^,^[32], and more likely to report casual partners[23]. Similarly, females in South Africa were more likely than males to report that their relationships were ‘ongoing’[32].

Early age at first sex among females was associated with 6-month point prevalence of concurrency. This is in line with broader research that has found early age at first sex to be associated with greater sexual risk behaviour later in life[31, 33] and highlights the importance of exploring sexual behaviour over the lifecourse[34, 35]. While the consistency of these findings support their validity, possible reporting bias cannot be ruled out, e.g. women generally are more likely to under-report casual partners or high partner number due to fear of stigma[19, 36–38], and women who honestly report an early age at first sex may also be most likely to honestly report multiple partners in the last year.

The point prevalence of concurrency reported by participants in this study (males 10.6%; females 2.3%) was higher than 20–24 year olds in the 2011/12 Tanzanian AIDS Indicator Survey (males 4.3%; females 1.0%), but lower than other settings in SSA. For example, a general population survey of 18–24 year olds in Botswana in 2007 found reported point prevalence of concurrency at the time of the survey to be 20% among both males and females[24]. In South Africa, a general population survey of 15–24 year olds in 2003 found the point prevalence of concurrency at the time of the survey to be 21.6% among males and 3.8% among females[22]. The 6 month point prevalence of concurrency in 15–49 year olds in national surveys in a number of SSA countries in 2009–11 ranged from 0.0–2.3% for females and 1.5–13.3% for males[39].

Many females in our sample reported having only one partner, usually spousal, within the past 12 months. This contrasts with the relatively high number of partners and concurrency reported by married males. Only 2% of married men reported having more than one wife, so most of their reported concurrency suggests extramarital relationships rather than polygyny. These findings highlight how married females may be at risk of contracting HIV from their husbands. The well-documented unequal power differentials in Tanzanian society[14, 18, 40] indicate that females are often not able to negotiate for use of condoms, or to refuse sex. In our study, many women and men reported having had sex with a partner when they did not want to. It is also possible, however, that female extramarital relationships may have been underreported due to fear of stigma. Participant observation and in-depth interviews in the 1999–2002 qualitative study found girls and young women commonly and substantially underreported their sexual partner numbers[18], but this was particularly pronounced for those who were married at the time of an interview. For example, many young women who reported sexual partners in premarital in-depth interviews subsequently denied they had ever had any partner other than their husband in interviews conducted 1–2 years later, after they had married.

Respondents in this study were asked to define their partners as spousal, other regular, casual, or sex worker. Each of those terms was open to variable interpretation in this study population. The wide disparity in the proportion of partners who are reported to be ‘spouses’ by males and females support qualitative findings in this population, which suggest that individuals within couples may perceive their relationship quite differently (often especially the woman seeing a husband when the man does not see a wife). This is compounded by the very informal nature of most marriages, which are most clearly identified as marriage when a couple move in together and cohabit, but can quickly become unclear outside of that category. The characteristics of the partnerships and partners in this study suggest a hierarchy of relationships, from spousal partner to sex worker, with partnership lengths decreasing, partner age difference narrowing, condom use increasing, frequency of sex decreasing, and confidence in the future of the partnership decreasing. It may be that the decision to describe a partner as ‘sex worker’, ‘casual’ or ‘regular’ is mainly a function of the length of time that the relationship has been ongoing, and that, for example, some long-term ‘casual’ partners become ‘regular’ partners over time, although qualitative research suggests that several other factors are important, including public knowledge of the relationship, and whether two partners live together at least part of the time[18].

Despite the limited nature of these categorisations, defining partnerships in this way may be useful, in the context of health services/research, to identify partnerships where higher risk sex is likely to occur. For HIV prevention program planning, a review of the extensive qualitative research on these topics within this population can provide a deeper understanding of the nature of the transition from ‘casual’ to ‘regular’ partner, the timing of cessation of use of condoms, and how frequency of sex changes over time [18, 41].

This survey took an unusual approach to measuring current relationships status by asking respondents both whether a relationship was ongoing and whether it had ended but the partners might have sex again in the future. This was based on qualitative findings that one of the most common sexual relationships among young people in the study population involved occasional, opportunistic sexual encounters within an open-ended timeframe[18]. Notably, 85% of ‘regular’ partnerships reported by females were ‘ongoing’, whereas, the corresponding proportion for males is 65%. It is possible that females are more optimistic about such relationships. However, these females in their early 20s had regular partners who were on average a couple of years older, whereas male respondents have regular partners who were on average 2 years younger, so it also may be that at an older age partnerships are more stable. The distribution of timing since last sex according to the reported relationship status, which was similar for both genders, seems plausible, and does not give any indication of over-optimistic reporting by females. Sexual behaviour is a sensitive issue, and multiple partnerships especially are something that both men and women might be motivated to under-report (men mainly due to fear of being caught by partners, women for that reason and also for fear of stigma or punishment). There is a strong possibility that these survey results under-represent women’s multiple partnerships.

Both main measures of concurrency—the UNAIDS-recommended 6-month point prevalence indicator, and the prevalence of current concurrency, based on the respondents’ assessment of whether the relationship was ‘ongoing’ at the time of the survey–had strengths and weaknesses. The UNAIDS indicator may offer greater clarity about whether a relationship has ended or not, but it also depends on accurate recall of earlier dates and relationship overlap. The current concurrency indicator may be more precise as a point in time estimate of relationships, but the respondent may be uncertain or incorrect about whether the relationship is ongoing or already has ended. Some authors have found reported current concurrency to be higher than reported 6-month point prevalence of concurrency possibly due to over optimism about the future of the relationship [27]. In this study, we found current and 6-month point prevalence of concurrency to be similar. We found that including relationships where the respondent reported that they ‘will’ and ‘might’ have sex again as “current” relationships might overestimate concurrency, but excluding those in the “relationship has ended but might have sex in the future” category might underestimate it (Table 2). We also found that the UNAIDS indicator led to a slight underestimation of concurrency, especially among males who reported >3 partners in the past year. Overall, the two indicators produced similar results. However, only 40–45% of respondents who were classified as having concurrent partners at either six months prior to the survey or at the time of the survey reported concurrent partners at both time points. This probably reflects how an individual’s relationships change over time, highlighting the limitations of both kinds of point prevalence estimates at the individual level. Nevertheless, at the population level, these measures of concurrency might be of more use, with current concurrency potentially easier to measure[42]. Some authors suggest that respondents should be asked directly about concurrency for each relationship, for example, when asking questions about partner X, an interviewer could ask the respondent whether s/he had other sexual partners while s/he was in a sexual relationship with partner X. There is some evidence that this direct approach leads to higher estimates of concurrency[42, 43].

The majority of males reporting multiple partnerships reported a mixture of regular and casual partners, and hence, the majority of concurrent partnerships were overlapping regular and casual partnerships (Table 2). However, of respondents who reported details on 2 or 3 partners in the past year, males reporting multiple regular partners were more likely to report concurrent partners at the 6-month time point prior to the survey than those reporting multiple casual or regular and casual partners. This is in keeping with data from South Africa where a concurrent relationship was usually a second ongoing partnership and not a ‘one-night stand’[22]. Elsewhere in South Africa, Malawi, and Tanzania, young people have also reported multiple partnerships of long duration [18, 22, 23, 26, 44]. This pattern is intrinsic to the nature of such partnerships, as the likelihood of a measure of concurrency capturing information on long-term, overlapping sexual relationships at any given point in time is higher than the likelihood of the measure collecting data on brief partnerships.

The triangulation of survey results and qualitative research findings from the same study population allowed for complex and meaningful analysis of difficult-to-research topics. However, the young people in our study were interviewed as a follow-up to a cluster-randomised trial of a primary school intervention, and as such youth who had not reached the last 3 years of primary school were not eligible to participate. Participants may have been less mobile and slightly more educated than the general population of young people. By design, we only collected data on up to 3 of the respondents’ most recent sexual partners in the previous year, and this limited our ability to adequately describe partnership patterns for the most sexually active respondents. Much of the analysis on concurrency was based on reported timing of first and last sex, which is subject to recall bias.

## Conclusions

This study provides one of the most comprehensive quantitative descriptions of partnership patterns of young people in an African setting. Young males, especially married men, reported high levels of multiple and concurrent partnerships. Far fewer young women reported multiple and concurrent partnerships, but we cannot definitively conclude that concurrency was uncommon for women, because stigma towards women’s multiple sexual partnerships might contribute to substantial under-reporting, as was found in extensive qualitative research in the study population. There needs to be continued effort to include men in HIV prevention campaigns, to encourage HIV testing and communication about HIV status within partnerships, fidelity within marriage and/or condoms in extramarital relationships, increased openness about young people’s sexual activity, and to empower women to protect themselves within partnerships [41]. Programmes should acknowledge that sex can be enjoyable and should be a positive experience for young people, while at the same time helping them to stay safe. Vague ‘be faithful’ slogans need to be improved upon and greater attention given to informing young people of the likely risk associated with different kinds of partnerships [45]. Health professionals need to be aware of high levels of multiple partnerships and concurrency when implementing contact tracing/treatment for treatable STIs and probe specifically for this in order to ensure all potentially infected individuals are treated. Interventions targeting delayed sexual onset also are important, particularly for girls, as early age at first sex was associated with greater reported concurrency later. Finally, male respondents reporting multiple regular partners were more likely to report concurrent partners than those who reported only casual partners, or a mixture of regular and casual partners. Measurement of length of relationship overlap, and exploration of its role in increasing HIV and other STI risk, warrant more in-depth examination.
